# Coil embolization of intralobar pulmonary sequestration - an alternative to surgery: a case report

**DOI:** 10.1186/s13256-018-1915-5

**Published:** 2018-12-21

**Authors:** John Ellis, Sumir Brahmbhatt, Daniel Desmond, Brian Ching, Jordanna Hostler

**Affiliations:** Tripler Army Medical Center, 1 Jarrett White Road, Honolulu, HI 96859 USA

**Keywords:** Intralobar, Pulmonary sequestration, Coil Embolization

## Abstract

**Background:**

Pulmonary sequestration is a congenital lung disease characterized by nonfunctioning pulmonary tissue that lacks normal communication with the bronchial tree and is supplied by a nonpulmonary systemic artery. Symptomatic bronchopulmonary sequestration is uncommon, seen more frequently in the pediatric population than in adults. It has traditionally been treated with surgical resection; however, a limited but growing number of cases have been treated with angiographic embolization. Given the inherent risks of cardiothoracic surgery, embolization of the anomalous vessel is an enticing alternative treatment. We present a case of a 56-year-old woman with known, symptomatic, intralobar pulmonary sequestration that was successfully treated with coil embolization.

**Case presentation:**

A 56-year-old Pacific Islander woman with a history of chronic myeloid leukemia was admitted to the hospital with an episode of hemoptysis. Computed tomography of the chest demonstrated left lower lobe intralobar pulmonary sequestration fed by a large tortuous vessel branching off of the descending thoracic aorta. Surgical resection of the sequestration is the current standard treatment strategy of symptomatic intralobar pulmonary sequestration. The cardiothoracic surgeon noted that given the size and location of arterial blood supply, intervention would involve thoracotomy and lobectomy. The interventional radiologist offered embolization of the lesion as an alternative to surgery. Multiple coils, 6–13 mm in size, were used to embolize the sequestration. No considerable flow distal to the coils was noted postembolization.

**Conclusions:**

Intralobar pulmonary sequestration is a rare condition that typically requires surgical management. This case demonstrates the efficacy of coil embolization as an alternative management strategy. To date, limited case reports of adults treated with endovascular embolization exist. Treatment of symptomatic pulmonary sequestration with embolization can be considered as an alternative to surgical resection.

## Background

Bronchopulmonary sequestration is an uncommon congenital lung malformation. Pulmonary sequestration (PS) is a congenital lung disease characterized by nonfunctioning pulmonary tissue that lacks normal communication with the bronchial tree and is supplied by a nonpulmonary systemic artery [1]. Only 0.15–6.4% of all cases of congenital lung malformation can be attributed to PS [2]. Patients generally become symptomatic early, and therefore PS is seen in the pediatric population more than in adults. Sixty percent of intralobar pulmonary sequestration (ILPS) is diagnosed before the age of 20, and it is seldom found in patients aged 40 years or older [1]. The current standard of care is surgical resection; however, a limited but growing number of cases have been treated with angiographic embolization. Given the inherent risks of cardiothoracic surgery, embolization of the anomalous vessel is an enticing alternative treatment. We present a case of a 56-year-old woman with known symptomatic ILPS that was successfully treated with coil embolization.

## Case presentation

A 56-year-old Pacific Islander woman was admitted to our hospital after she presented with hemoptysis, which she quantified as about a handful. She was a lifelong nonsmoker with no history of obstructive or restrictive lung disease and no reported allergies. Her past medical history was significant for chronic myeloid leukemia on imatinib therapy and a previous case of mild hemoptysis 6 years prior to current presentation. At that time, the patient was diagnosed with ILPS; however, her symptoms resolved, and she did not pursue any treatment.

**Fig. 1:**
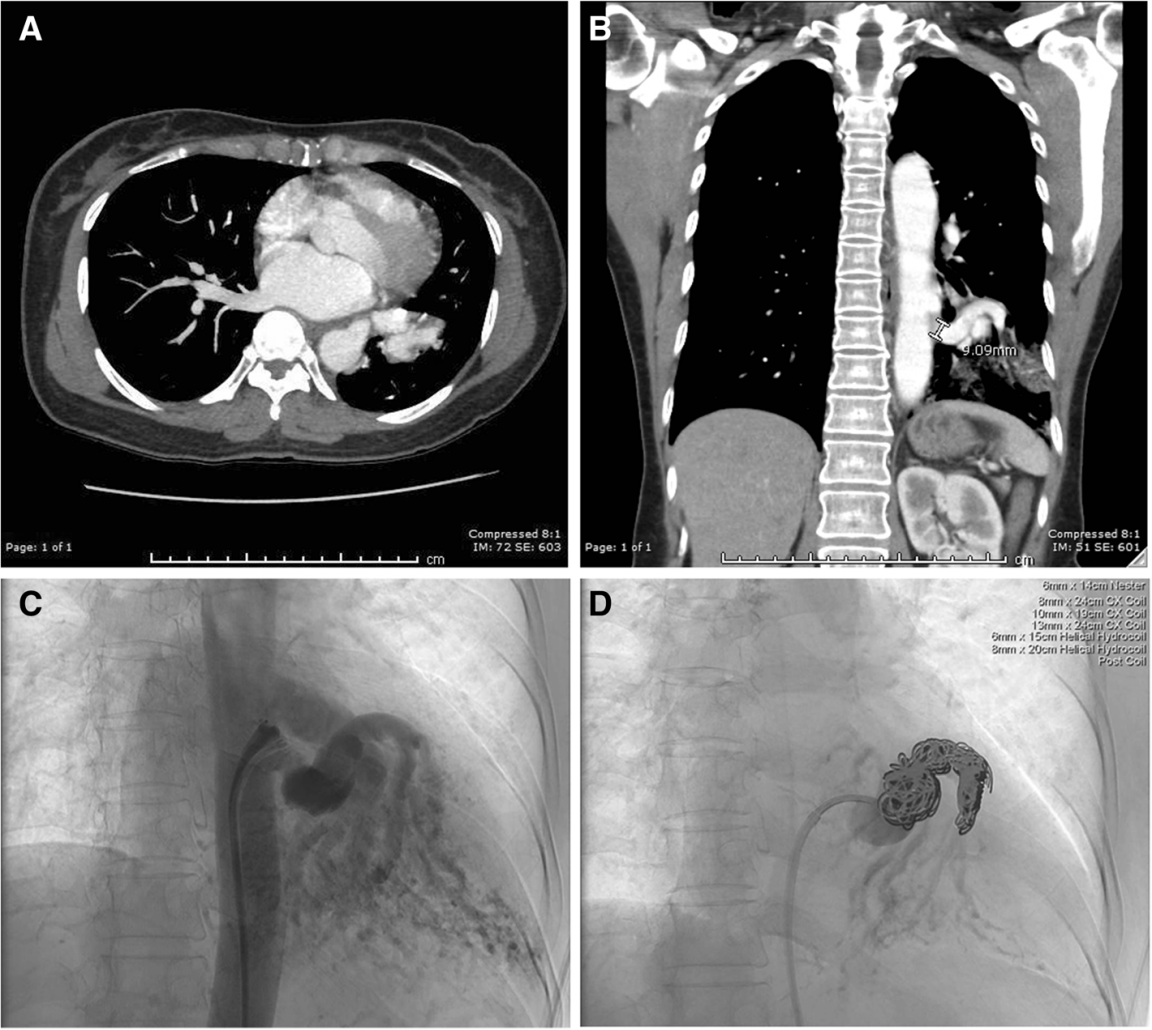
**a** Axial computed tomographic scan with contrast showing a tortuous vessel that leads to a focal area of mixed consolidation and ground glass representing intralobar pulmonary sequestration. **b** Coronal view showing the origin of the aberrant vessel, which measures approximately 1 cm. **c** Angiography of vessel prior to embolization. **d** Postembolization angiogram showing coils in place with no considerable flow

On arrival, the patient was hemodynamically stable (blood pressure 100/60 mmHg, heart rate 54 beats/minute) with mild anemia (hemoglobin 12.0 g/dl). Her physical examination was notable for coarse breath sounds throughout the lower left lung field without dullness to percussion to suggest hemothorax. Her cardiac, abdominal, and neurological examinations were without focal findings. Her airway was patent, and her oral mucosa was moist. Her laboratory work was notable only for the mild anemia noted above; her chemistry panel and coagulation profiles were within normal limits. Her body mass index was 20.8 kg/m^2^. A chest x-ray showed left lower lobe nodular opacities. Computed tomography of the chest with contrast demonstrated left lower lobe ILPS. The aberrant vessel was traced to its origin at the descending thoracic aorta, where it measured approximately 1 cm (Fig. 1a, b). Bronchoscopy was not pursued, because this could induce coughing and/or dislodge a clot. Furthermore, with radiographic evidence of the sequestration, another source of bleeding was not clinically suspected.

With PS, the usual treatment is resection of the sequestration. In those patients with the extralobar subtype, this is completed by removal of only the sequestration. The intralobar type is managed by segmental resection or lobectomy [3]. The patient was evaluated by a cardiothoracic surgeon, who noted that surgical resection would likely require a thoracotomy and lobectomy instead of a less invasive video-assisted thoracoscopic surgery (VATS), given the size and location of the arterial blood supply to the sequestration. When we explained the risks and benefits to the patient, she declined surgery, given her ongoing treatment for chronic myeloid leukemia and her personal desire to avoid surgery.

After review of the case with a multidisciplinary team, the interventional radiology service offered embolization of the lesion as an alternative to surgery. Multiple 6–13-mm coils, including Nester Embolization Coils (Cook Medical, Bloomington, IN, USA), AZUR® CX Peripheral Coil System (Terumo Interventional Systems, Somerset, NJ, USA), and hydrocoils, were used to embolize the sequestration. There was no considerable flow distal to the coils postembolization (Fig. 1c, d). The patient’s postprocedural course was notable for pleurisy that responded to oral analgesia. No signs or symptoms of infection occurred, and the patient did not require antibiotics. At her 9-month and 1-year follow-up visits, she reported no pulmonary symptoms, cough, or hemoptysis.

## Discussion

This case demonstrates a successful alternative treatment for symptomatic ILPS in an adult. The patient had hemoptysis that required attention; however, given her personal desire to avoid surgery, coil embolization was successfully used for treatment. This case provides an opportunity to discuss the background of this rare lung malformation and treatment options when it presents in an adult population.

Bronchopulmonary sequestration is an uncommon congenital lung malformation; only 0.15–6.4% of all cases of congenital lung malformation can be attributed to PS [1]. Symptoms typically present early, and therefore it is seen in the pediatric population more than in adults. It has been reported that 60% of ILPS is diagnosed before the age of 20, and it is seldom found in patients aged 40 years or older [1]. Because of the nonspecific symptoms, it is often difficult to diagnose without direct investigation, and imaging of the vasculature and pulmonary parenchyma is done [4].

PS is a congenital lung disease characterized by nonfunctioning pulmonary tissue that lacks normal communication with the bronchial tree and is supplied by a nonpulmonary systemic artery [5]. Although various systems have been used to classify PS, including the Pryce classification, sequestration spectrum, and pulmonary malinosculation spectrum [6], for our purposes, we will classify PS anatomically. It has two main subtypes: intralobar, which is inside the lung lobe and lacks its own pleura, and extralobar pulmonary, which is outside the lung lobe and has its own visceral pleura [7]. Our patient’s case is of the intralobar variant, which represents 75% of PS [8, 9].

Cough, sputum production, and recurrent episodes of pneumonia are the most common symptoms of patients with PS [10]. With the intralobar variant, half of all patients reach the age of 20 years before being diagnosed, in contrast to the extralobar variant, which is more commonly diagnosed in the pediatric population [11]. In 5–15% of the cases, patients are asymptomatic, and the anomaly is discovered coincidently [12]. Minor hemoptysis, often occurring with infection, is common. More severe hemoptysis is possible, and cases of bleeds into the pleural space, the esophagus, and the sequestration tissue have been described, with some of these cases being fatal [13, 14]. Hemoptysis is thought to be secondary to high-pressure blood flow in the sequestered lung from the anomalous systemic arteries [15].

The current standard of care for PS is surgical excision. Segmentectomy, either as a wedge resection or as anatomical segmentectomy, is the operative choice for symptomatic cases of extralobar PS and ILPS. For symptomatic cases where segmentectomy is not possible owing to size/location, a lobectomy is required [16–18]. This has been well described in the literature and is considered to be curative. Resection of the extralobar variant is considered easier because the anomalous mass has its own pleura, and commonly a segmentectomy is sufficient. This is in contrast to the intralobar type, which has the same pleura as the remaining lung and more often requires lobectomy [19, 20]; however, parenchymal preservation is always the goal. Surgical approaches can be open or done thoracoscopically. Specifically, VATS is increasingly used with good results [21, 22]. Generally, resection has good outcomes and can be carried out safely; however, risk of complication is inherent in this invasive procedure [23].

Endovascular embolization of PS is an attractive minimally invasive option. When compared with conventional surgery, it is potentially less prone to associated complications. Treatment of PS with endovascular embolization is described in the pediatric literature [24, 25]; however, there is limited experience in adults [26–29].

The literature documenting the side effects of embolization appears to be more robust in the pediatric population. Infection, thrombosis at puncture sites, fever, pain, hypertension, and migration of embolization material to nontarget arteries have been reported [30]. In adults, the described sequelae largely mirror the side effects described in a similar procedure, such as adult embolization of arterial venous malformations in pulmonary parenchyma and embolization of bronchial arteries in cases of massive hemoptysis [31]. The most feared complication is inadvertent embolization of a spinal artery, which has been reported in cases of bronchial artery embolization [32]. However, retained nonaerated pulmonary parenchymal tissue as a result of embolization is also feared because this would create a difficult-to-access nidus for infection [33]. A large-scale comparison of surgical vs. endovascular treatments in adults has not been published to date. This case report adds to the growing number of reports of adult patients who have been treated with embolization, thus adding to a population that can be compared with those receiving surgical treatment.

## Conclusions

Treatment of symptomatic PS with embolization can be considered as an alternative to surgical resection in cases where surgery would have significant morbidity and mortality risks. Currently, no comprehensive studies have been completed to compare standard care (that is, surgical excision) with embolization. A multidisciplinary team should assess the patient to determine which treatment course provides the best risk–reward balance and likelihood of a durable response.

## Abbreviations

ILPS: Intralobar pulmonary sequestration
PS: Pulmonary sequestration
VATS: Video-assisted thoracoscopic surgery
